# Causal relationship between gut microbiota and differentiated thyroid cancer: a two-sample Mendelian randomization study

**DOI:** 10.3389/fonc.2024.1375525

**Published:** 2024-04-26

**Authors:** Shaojun Hu, Chuangang Tang, Ling Wang, Fang Feng, Xiaoxin Li, Mingyu Sun, Lijun Yao

**Affiliations:** ^1^ Department of Oncology, Suzhou Ninth People’s Hospital, Suzhou Ninth Hospital Affiliated to Soochow University, Suzhou, China; ^2^ Department of Breast Surgery, Xuzhou Central Hospital, The Affiliated Xuzhou Hospital of Medical College of Southeast University, Xuzhou, China; ^3^ Department of Critical Care Medicine, The People’s Hospital of Huaiyin, Jinan, China; ^4^ Department of Pathology, Xuzhou Central Hospital, The Affiliated Xuzhou Hospital of Medical College of Southeast University, Xuzhou, China

**Keywords:** causality, gut microbiota, differentiated thyroid cancer, Mendelian randomization, genome-wide association study

## Abstract

**Background:**

The gut microbiota has been significantly associated with differentiated thyroid cancer (DTC). However, the causal relationship between the gut microbiota and DTC remains unexplored.

**Methods:**

Genome-wide association study (GWAS) summary databases were utilized to select exposures and outcomes. The Mendelian randomization (MR) method was employed to investigate the causal relationship between the gut microbiota and DTC. A sensitivity analysis was performed to assess the reliability of the findings.

**Results:**

Four bacterial traits were associated with the risk of DTC: Class Mollicutes [odds ratio (OR) = 10.953, 95% confidence interval (95% CI): 2.333–51.428, *p* = 0.002], Phylum Tenericutes (OR = 10.953, 95% CI: 2.333–51.428, *p* = 0.002), Genus Eggerthella (OR = 3.219, 95% CI: 1.033–10.024, *p* = 0.044), and Order Rhodospirillales (OR = 2.829, 95% CI: 1.096–7.299, *p* = 0.032). The large 95% CI range for the Class Mollicutes and the Phylum Tenericutes may be attributed to the small sample size. Additionally, four other bacterial traits were negatively associated with DTC: Genus Eubacterium fissicatena group (OR = 0.381, 95% CI: 0.148–0.979, *p* = 0.045), Genus Lachnospiraceae UCG008 (OR = 0.317, 95% CI: 0.125–0.801, *p* = 0.015), Genus Christensenellaceae R-7 group (OR = 0.134, 95% CI: 0.020–0.886, *p* = 0.037), and Genus Escherichia Shigella (OR = 0.170, 95% CI: 0.037–0.769, *p* = 0.021).

**Conclusion:**

These findings contribute to our understanding of the pathological mechanisms underlying DTC and provide novel insights for the clinical treatment of DTC.

## Introduction

Thyroid cancer is the most prevalent malignancy of the endocrine system (1). According to the GLOBOCAN (2020) database, there were 586,202 new cases of thyroid cancer in 2020 worldwide, constituting 3.0% of all cancer incidences (2, 3). Thyroid cancer encompasses four primary pathological classifications: papillary thyroid cancer (PTC), follicular thyroid cancer (FTC), medullary thyroid cancer (MTC), and anaplastic thyroid carcinoma (ATC) (4, 5). Differentiated thyroid cancer (DTC), comprising PTC and FTC, accounts for most thyroid cancer cases and typically presents a favorable prognosis (6). However, DTC is susceptible to local lymph node metastasis, contributing to a recurrence rate of up to 20% within 10 years (7). Certain subtypes, such as the diffuse sclerosing variant (DSV), exhibit relatively high invasiveness and are associated with a dismal prognosis (8). Therefore, a profound comprehension of the mechanisms underlying the onset and development of DTC is imperative.

Accumulating evidence suggests that the gut microbiota plays a crucial role in malignant tumors, including colorectal cancer, lung cancer, and breast cancer (9–11). Yu et al. (12) observed a significant decline in the richness and diversity of the gut microbiota in patients with DTC compared to healthy individuals. They developed a 10-genus microbial signature capable of effectively distinguishing patients with DTC from healthy individuals. Moreover, the gut microbiota is closely associated with the therapeutic response to radioactive iodine (RAI) following thyroidectomy (13). Before RAI treatment, thyroid hormone withdrawal (THW) is typically necessary to stimulate the secretion of thyroid stimulating hormone; however, THW-related complications significantly decrease quality of life. A recent randomized clinical trial showed that probiotics improve multiple symptoms induced by THW, including constipation and excessive weight gain (14). These findings indicate the involvement of the gut microbiota in the progression of DTC. Nevertheless, the causal relationship between the gut microbiota and DTC remains unexplored.

In the present study, the Mendelian randomization (MR) method was employed to ascertain whether there exists a causal relationship between the gut microbiota and DTC. Our findings may provide crucial insights into the pathological mechanisms underlying DTC.

## Patients and methods

### Patient data

A genome-wide association study (GWAS) dataset of the gut microbiota was downloaded from MiBioGen (https://mibiogen.gcc.rug.nl/menu/main/home/) and utilized as an exposure variable. To investigate the interactive effects of human genetics and gut microbiota, 16S rRNA gene sequencing was performed on 18,340 individuals from 24 different cohorts. Following the exclusion of entries with unknown taxonomic information, a total of 196 bacterial traits were selected, comprising 9 phyla, 16 classes, 20 orders, 32 families, and 119 genera. A GWAS dataset of DTC (GWAS ID: ieu-a-1082) was obtained from the IEU OpenGWAS project (https://gwas.mrcieu.ac.uk/) and employed as the outcome variable (15). Initially, a total of 701 Italian individuals with DTC (median age, 46 years) were included, of whom 649 remained after rigorous quality control measures. All cases were histologically validated as DTC, without further differentiation between PTC and FTC subtypes.

To establish a definitive causality between the gut microbiota and DTC, we employed the following screening criteria for instrumental variables (IVs): 1) single-nucleotide polymorphisms (SNPs) had a strong correlation with exposure (gut microbiota). The level of significance (*p*-value) was set to *p* < 1 × 10^−5^, consistent with the established protocol in prior studies (16, 17). 2) An F-statistic threshold >10 was utilized to mitigate weak IV bias. 3) SNPs exhibiting linkage disequilibrium effects were excluded, employing an R^2^ cutoff of 0.001 and a clumping window size of 10,000 kb. 4) In cases where SNPs were absent in the outcome, proxy SNPs (R^2^ > 0.8) were obtained from the 1000 Genomes Project (http://www.internationalgenome.org/). 5) The threshold for allele frequencies was set to 0.01. The study was approved by the Ethics Committee of Suzhou Ninth Hospital Affiliated to Soochow University.

### Statistical analysis

Before conducting MR analysis, palindromic SNPs were excluded to harmonize the effects of the SNPs, and Steiger filtering was performed to ensure the correct directionality of each SNP. Five common MR methods were utilized in this study: inverse variance weighted (IVW), weighted median, weighted mode, simple mode, and MR-Egger methods. The IVW method estimates causal relationships by integrating the effect sizes of multiple genetic variations and applying inverse variance weighting. This approach enhances statistical power, mitigates estimation bias, and improves the accuracy of causal relationship assessment, making it a preferred choice in MR-related studies. The odds ratio (OR) and 95% confidence interval (95% CI) were determined. The MR-Egger intercept test was employed to assess directional horizontal pleiotropy (18). Cochran’s Q statistic was used to assess the heterogeneity in the data (19). MR Pleiotropy Residual Sum and Outlier (MR-PRESSO) analysis was performed to assess the presence of outliers (20). All statistical analyses were conducted using R software (version 4.3.1), primarily utilizing the R packages “TwoSampleMR” (version 0.5.7), “MRPRESSO” (version 1.0), “ieugwasr” (version 0.1.5), and “plinkbinr” (version 0.0.0.9000). A *p*-value <0.05 indicated statistical significance.

## Results

### MR analysis

**Figure 1:**
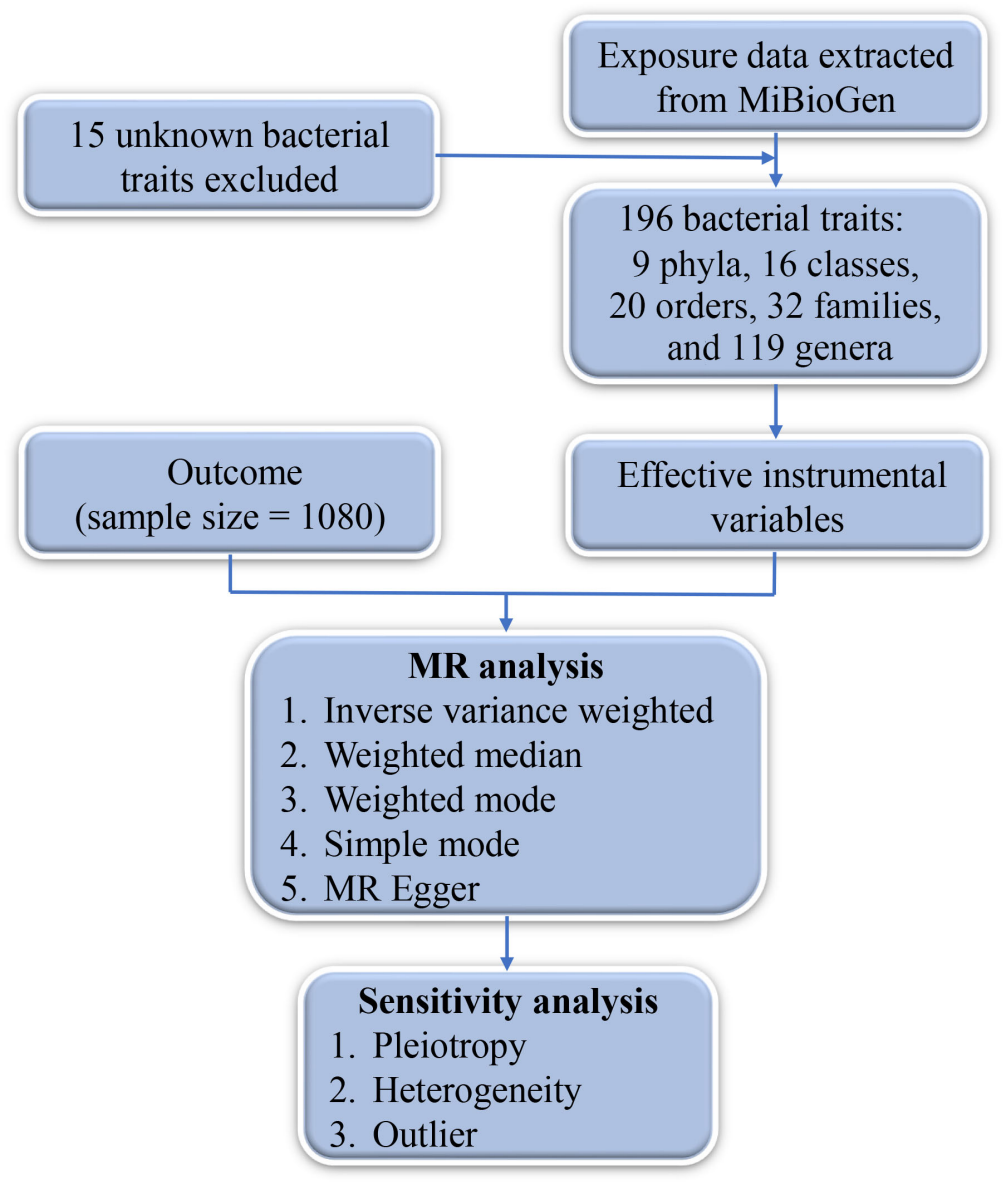
Flowchart depicting the MR analysis. MR, Mendelian randomization.

**Figure 2:**
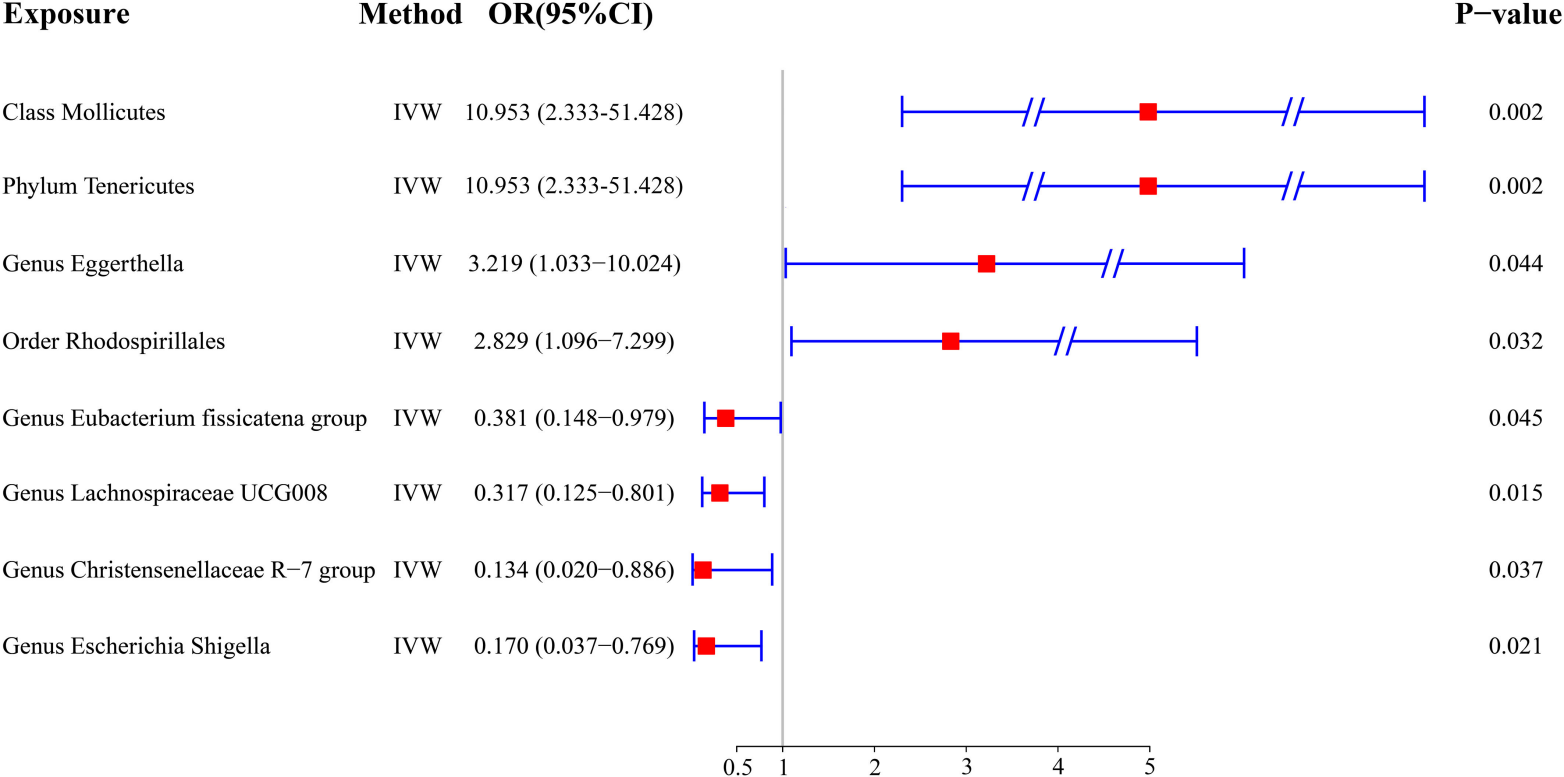
A forest plot illustrating the MR results. MR, Mendelian randomization.

**Figure 3:**
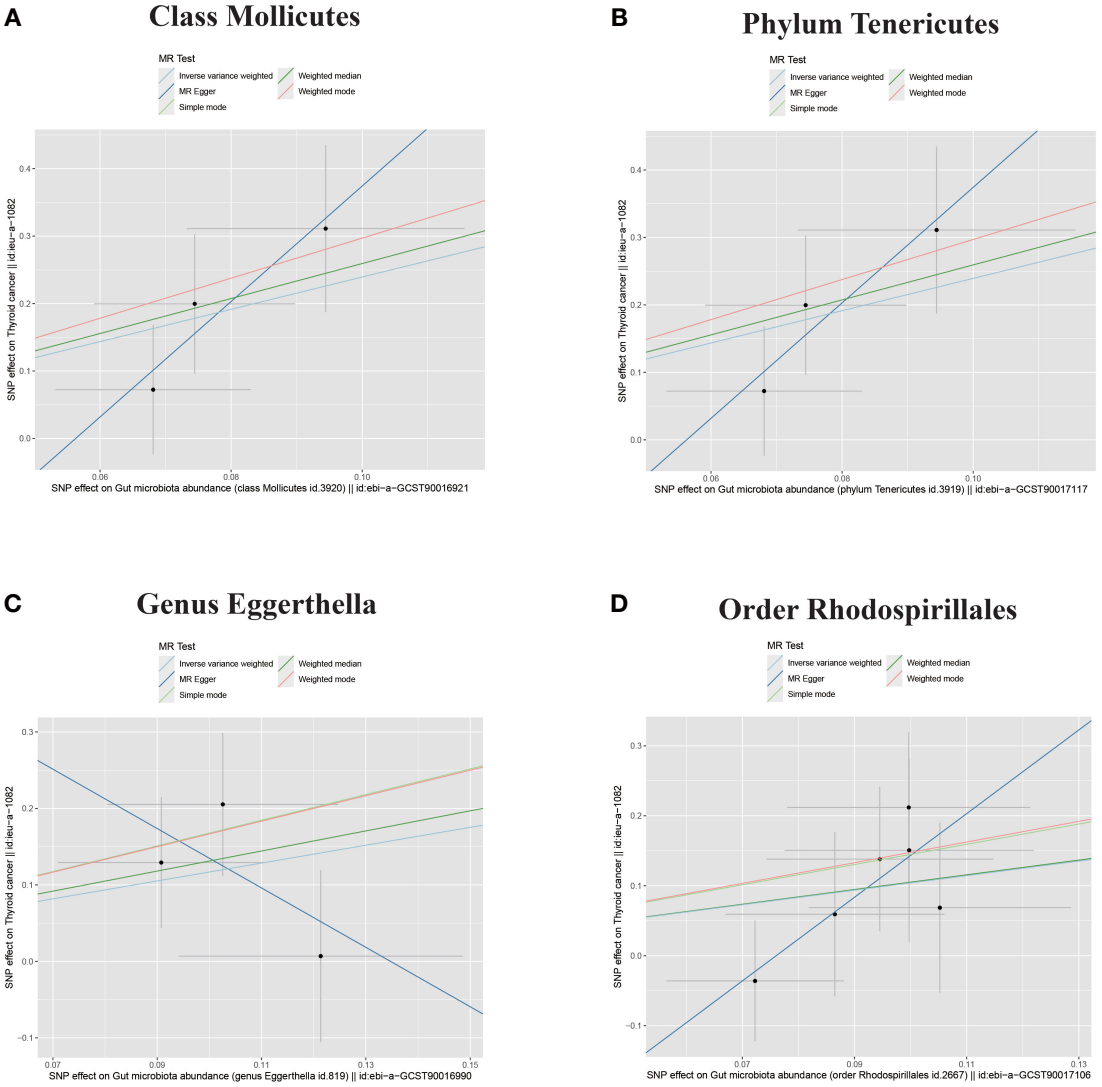
Scatter plots of four bacterial traits positively associated with DTC. **(A)** Class Mollicutes. **(B)** Phylum Tenericutes. **(C)** Genus Eggerthella. **(D)** Order Rhodospirillales. DTC, differentiated thyroid cancer; MR, Mendelian randomization; SNP, single-nucleotide polymorphism.

The outcome comprised a total of 1,080 individuals, including 649 DTC cases and 431 controls (**Figure 1**). The IVW analysis showed that the Class Mollicutes and the Phylum Tenericutes were positively correlated with DTC, suggesting that Mollicutes and Tenericutes are associated with an increased risk of DTC (**Figures 2**, **3A, B**, **Table 1**; Class Mollicutes, OR = 10.953, 95% CI: 2.333–51.428, *p* = 0.002; Phylum Tenericutes, OR = 10.953, 95% CI: 2.333–51.428, *p* = 0.002). The risk effects associated with both entities on DTC were nearly identical, as the Class Mollicutes falls within the Phylum Tenericutes. The weighted median method further confirmed the results (**Table 1**; Class Mollicutes, OR = 13.375, 95% CI: 1.731–103.376, *p* = 0.013; Phylum Tenericutes, OR = 13.375, 95% CI: 1.730–103.393, *p* = 0.013). In addition, no directional horizontal pleiotropy was observed (**Supplementary Table 1**; Class Mollicutes, Egger intercept = −0.482, *p* = 0.482; Phylum Tenericutes, Egger intercept = −0.482, *p* = 0.482). Furthermore, Cochran’s Q test indicated no heterogeneity in individual causal effects (**Supplementary Table 2**; Class Mollicutes, Cochran’s Q = 0.290, *p* = 0.590; Phylum Tenericutes, Cochran’s Q = 0.290, *p* = 0.590). Additionally, the MR-PRESSO analysis did not identify any potential outliers (**Supplementary Table 3**).

**Table 1:** Causal effects of gut microbiota on DTC.

Exposure	No. of SNPs	Methods	Beta	SE	OR (95% CI)	*p*-Value
Class Mollicutes	3	Inverse variance weighted	2.394	0.789	10.953 (2.333–51.428)	0.002
	3	Weighted median	2.593	1.043	13.375 (1.731–103.376)	0.013
	3	Weighted mode	2.970	1.310	19.491 (1.494–254.263)	0.152
	3	Simple mode	2.970	1.345	19.491 (1.395–272.254)	0.158
	3	MR-Egger	8.559	5.872	5211.449 (0.052–5.19e+8)	0.383
Phylum Tenericutes	3	Inverse variance weighted	2.394	0.789	10.953 (2.333–51.428)	0.002
	3	Weighted median	2.593	1.043	13.375 (1.730–103.393)	0.013
	3	Weighted mode	2.970	1.286	19.491 (1.568–242.297)	0.147
	3	Simple mode	2.970	1.324	19.491 (1.454–261.229)	0.154
	3	MR-Egger	8.559	5.872	5211.45 (0.052–5.19e+8)	0.383
Genus Eggerthella	3	Inverse variance weighted	1.169	0.580	3.219 (1.033–10.024)	0.044
	3	Weighted median	1.313	0.729	3.716 (0.891–15.503)	0.072
	3	Simple mode	1.678	0.938	5.354 (0.852–33.653)	0.216
	3	Weighted mode	1.667	0.935	5.295 (0.847–33.084)	0.217
	3	MR-Egger	−3.883	4.933	0.021 (0–525.661)	0.575
Order Rhodospirillales	6	Inverse variance weighted	1.040	0.484	2.829 (1.096–7.299)	0.032
	6	Weighted median	1.051	0.622	2.861 (0.845–9.688)	0.091
	6	MR-Egger	5.979	3.674	395.189 (0.295–529391.600)	0.179
	6	Simple mode	1.448	0.931	4.253 (0.686–26.364)	0.181
	6	Weighted mode	1.477	0.982	4.381 (0.640–29.996)	0.193
Genus Eubacterium fissicatena group	4	Inverse variance weighted	−0.966	0.482	0.381 (0.148–0.979)	0.045
	4	Weighted median	−1.133	0.530	0.322 (0.114–0.910)	0.033
	4	Weighted mode	−1.320	0.651	0.267 (0.075–0.956)	0.136
	4	Simple mode	−1.320	0.754	0.267 (0.061–1.171)	0.178
	4	MR-Egger	0.332	4.317	1.394 (0–6586.220)	0.946
Genus Lachnospiraceae UCG008	5	Inverse variance weighted	−1.150	0.473	0.317 (0.125–0.801)	0.015
	5	Weighted median	−1.328	0.628	0.265 (0.077–0.907)	0.034
	5	Simple mode	−1.467	0.743	0.231 (0.054–0.990)	0.120
	5	Weighted mode	−1.479	0.810	0.228 (0.047–1.114)	0.142
	5	MR-Egger	1.517	4.498	4.560 (0.001–30754.110)	0.758
Genus Christensenellaceae R-7 group	3	Inverse variance weighted	−2.010	0.964	0.134 (0.020–0.886)	0.037
	3	Weighted median	−1.746	1.232	0.174 (0.016–1.950)	0.156
	3	Weighted mode	−1.598	1.271	0.202 (0.017–2.444)	0.336
	3	Simple mode	−1.598	1.401	0.202 (0.013–3.149)	0.372
	3	MR-Egger	−2.934	2.952	0.053 (0–17.299)	0.502
Genus Escherichia Shigella	3	Inverse variance weighted	−1.774	0.772	0.170 (0.037–0.769)	0.021
	3	Weighted median	−1.874	0.971	0.154 (0.023–1.030)	0.054
	3	Simple mode	−1.968	1.074	0.140 (0.017–1.147)	0.208
	3	Weighted mode	−1.960	1.121	0.141 (0.016–1.267)	0.222
	3	MR-Egger	−2.193	3.434	0.112 (0–93.381)	0.638

SNP, single-nucleotide polymorphism; SE, standard error; OR, odds ratio; 95% CI, 95% confidence interval; DTC, differentiated thyroid cancer.

IVW analysis identified the Genus Eggerthella and the Order Rhodospirillales as risk factors for DTC (**Figures 2**, **3C, D**, **Table 1**; Genus Eggerthella, OR = 3.219, 95% CI: 1.033–10.024, *p* = 0.044; Order Rhodospirillales, OR = 2.829, 95% CI: 1.096–7.299, *p* = 0.032). However, these results lacked support from the weighted median method (**Table 1**; Genus Eggerthella, OR = 3.716, 95% CI: 0.891–15.503, *p* = 0.072; Order Rhodospirillales, OR = 2.861, 95% CI: 0.845–9.688, *p* = 0.091). MR-Egger analysis indicated no directional horizontal pleiotropy (Genus Eggerthella, Egger intercept = 0.523, *p* = 0.490; Order Rhodospirillales, Egger intercept = −0.455, *p* = 0.246). Cochran’s Q test revealed no heterogeneity in individual causal effects (**Supplementary Table 2**; Genus Eggerthella, Cochran’s Q = 1.139, *p* = 0.286; Order Rhodospirillales, Cochran’s Q = 1.288, *p* = 0.863).

**Figure 4:**
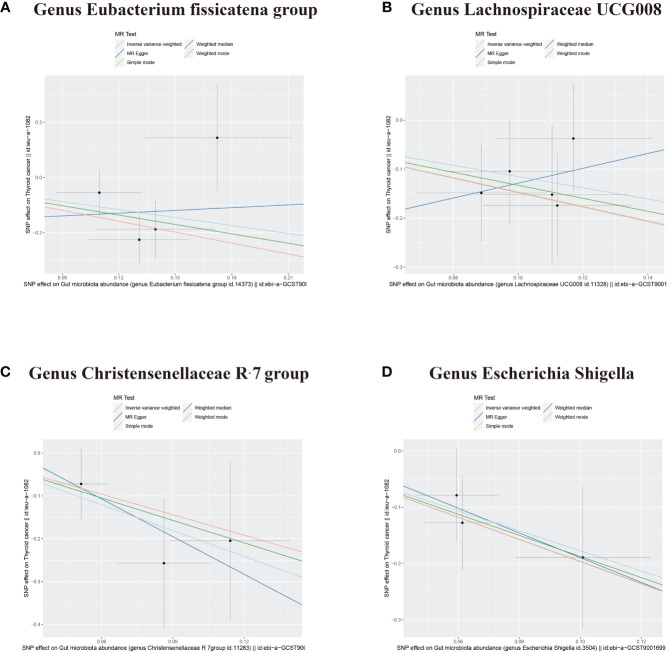
Scatter plots depicting four bacterial traits negatively associated with DTC. **(A)** Genus Eubacterium fissicatena group. **(B)** Genus Lachnospiraceae UCG008. **(C)** Genus Christensenellaceae R-7 group. **(D)** Genus Escherichia Shigella. DTC, differentiated thyroid cancer; MR, Mendelian randomization; SNP, single-nucleotide polymorphism.

In addition to the gut microbiota associated with an increased risk of DTC, the microbiota protecting against DTC was identified. IVW analysis revealed that the Genus Eubacterium fissicatena group and the Genus Lachnospiraceae UCG008 were negatively correlated with DTC, indicating their potential protective roles against DTC (**Figures 2**, **4A, B**, **Table 1**; Genus Eubacterium fissicatena group, OR = 0.381, 95% CI: 0.148–0.979, *p* = 0.045; Genus Lachnospiraceae UCG008, OR = 0.317, 95% CI: 0.125–0.801, *p* = 0.015). These results were further supported by the weighted median analysis (**Table 1**; Genus Eubacterium fissicatena group, OR = 0.322, 95% CI: 0.114–0.910, *p* = 0.033; Genus Lachnospiraceae UCG008, OR = 0.265, 95% CI: 0.077–0.907, *p* = 0.034). In addition, no directional horizontal pleiotropy was observed (**Supplementary Table 1**; Genus Eubacterium fissicatena group, Egger intercept = −0.169, *p* = 0.790; Genus Lachnospiraceae UCG008, Egger intercept = −0.280, *p* = 0.593). Cochran’s Q test indicated no heterogeneity in the individual causal effects (**Supplementary Table 2**; Genus Eubacterium fissicatena group, Cochran’s Q = 4.218, *p* = 0.121; Genus Lachnospiraceae UCG008, Cochran’s Q = 0.860, *p* = 0.835).

The Genus Christensenellaceae R-7 group and the Genus Escherichia Shigella emerged as protective factors against DTC, as revealed by the IVW analysis (**Figures 2**, **4C, D**, **Table 1**; Genus Christensenellaceae R-7 group, OR = 0.134, 95% CI: 0.020–0.886, *p* = 0.037; Genus Escherichia Shigella, OR = 0.170, 95% CI: 0.037–0.769, *p* = 0.021). However, these results were not supported by the weighted median analysis (**Table 1**; Genus Christensenellaceae R-7 group, OR = 0.174, 95% CI: 0.016–1.950, *p* = 0.156; Genus Escherichia Shigella, OR = 0.154, 95% CI: 0.023–1.030, *p* = 0.054). Moreover, the MR-Egger analysis indicated no directional horizontal pleiotropy (**Supplementary Table 1**; Genus Christensenellaceae R-7 group, Egger intercept = 0.069, *p* = 0.796; Genus Escherichia Shigella, Egger intercept = 0.030, *p* = 0.921). Additionally, Cochran’s Q test revealed no heterogeneity in individual causal effects (**Supplementary Table 2**; Genus Christensenellaceae R-7 group, Cochran’s Q = 0.353, *p* = 0.552; Genus Escherichia Shigella, Cochran’s Q = 0.142, *p* = 0.707).

## Discussion

Most studies on the gut microbiota have predominantly focused on malignant tumors of the digestive tract (21). Gradually, it has been acknowledged that the gut microbiota also impacts non-gastrointestinal tumors through various mechanisms, including inflammation and immunoregulation, metabolic pathways, and bacterial translocation. For example, *Bacteroides fragilis* induces the differentiation of Treg cells, thereby promoting the formation of an immunosuppressive microenvironment through the production of immunosuppressive factors such as IL-10 and TGF-β, ultimately contributing to the development of gliomas (22, 23). Additionally, *Ruminococcus* sp. DSM_100440 has been found to convert androgen precursors into androgens, expediting the progression of castration-resistant prostate cancer (CRPC) (24). In this study, we identified eight bacterial traits significantly associated with DTC. Among these, four bacterial traits (Class Mollicutes, Phylum Tenericutes, Genus Eggerthella, and Order Rhodospirillales) were associated with the risk of DTC, while the remaining four traits (Genus Eubacterium fissicatena group, Genus Lachnospiraceae UCG008, Genus Christensenellaceae R-7 group, and Genus Escherichia Shigella) exhibited a protective effect against DTC. These findings contribute to our comprehension of the role of the gut microbiota in non-gastrointestinal tumors and offer a novel avenue for DTC treatment.

Christensenellaceae is widely distributed throughout the digestive tract and is intricately linked to human health (25). We found that the Genus Christensenellaceae R-7 group had a significant negative causality with DTC. Consistent with our findings, Lu et al. showed that the Genus Christensenellaceae R-7 group is significantly associated with DTC (26). They suggested that the Genus Christensenellaceae R-7 group may promote the occurrence and development of DTC by regulating lipid metabolism, as indicated by the marked inhibition of lipid digestion and steroid biosynthesis pathways. Similarly, a reduced abundance of the Genus Christensenellaceae R-7 group has been observed in patients with colorectal cancer (27), further underscoring the potential of this genus as a probiotic.

The Family Lachnospiraceae has been reported to play a protective role against colorectal carcinogenesis by bolstering the tumor immunosurveillance function of CD8^+^ T cells (28). Additionally, it has been demonstrated to serve a protective role in the regulation of radiation-induced intestinal damage (29). However, Zheng et al. (13) elucidated a greater abundance of the Family Lachnospiraceae in patients with DTC exhibiting a non-excellent response to RAI compared to those exhibiting an excellent response. We speculate that the absence of further taxonomic classification within the Lachnospiraceae family may have contributed to these discrepant findings. A genus-level analysis showed that the abundance of the Genus Lachnospiraceae UCG010 is significantly higher in patients with DTC exhibiting an excellent response to RAI than in those exhibiting a non-excellent response, suggesting that the Genus Lachnospiraceae UCG010 is a protective factor. Similarly, our analysis indicated that Lachnospiraceae UCG008 exerts protective effects against DTC.

The abundance of Mollicutes/Tenericutes was significantly increased in various tumor tissues, including gastric and lung cancers (30, 31). Employing animal models, Lee et al. (32) elucidated a positive correlation between gut Mollicutes/Tenericutes and tumor burden in colitis-associated cancer. To the best of our knowledge, our study is the first to demonstrate the causal relationship between gut Mollicutes/Tenericutes and DTC. The large 95% CI range observed for the Class Mollicutes and Phylum Tenericutes may be attributed to the small sample size. Further studies are warranted to elucidate the pathogenic mechanisms of Mollicutes/Tenericutes.

This study has a few limitations. First, it included only one cohort comprising 1,080 participants, potentially resulting in a limited number of valid SNPs. Studies involving larger cohorts are necessary to establish more robust causal links between the gut microbiota and DTC. Second, although taxonomic classification has identified approximately 1,000 species of gut microbiota (33), our analysis was limited to 119 genera, precluding consideration at the species level. Third, due to the small sample size, DTC was treated as a single entity in this study. In fact, DTC can be classified into various subtypes, each exhibiting distinct biological behaviors. In future studies, histopathological subclassifications will be pivotal for refining precision treatments for DTC. Fourth, potential unaddressed confounding factors could impact the accuracy and generalizability of our findings. Finally, discrepancies observed across MR methods raise concerns regarding the robustness of certain associations, while the lack of functional insights leaves unanswered questions regarding biological mechanisms.

In summary, our investigation revealed that eight bacterial traits exert a significant causal effect on DTC. These findings enhance our comprehension of the pathological mechanisms underlying DTC and provide a novel avenue for its treatment.

## Data availability statement

The original contributions presented in the study are included in the article/**Supplementary Material**. Further inquiries can be directed to the corresponding authors.

## Ethical statement

The experiments were ethically approved by the Suzhou Ninth People’s Hospital.

## Ethics statement

The studies involving humans were approved by the Ethics Committee of Suzhou Ninth Hospital Affiliated to Soochow University. The studies were conducted in accordance with the local legislation and institutional requirements. Written informed consent for participation was not required from the participants or the participants’ legal guardians/next of kin in accordance with the national legislation and institutional requirements.

## Author contributions

SH: Data curation, Formal analysis, Methodology, Validation, Software, Writing – original draft. FF: Conceptualization, Data curation, Formal analysis, Investigation, Writing – original draft. CT: Data curation, Investigation, Methodology, Resources, Validation, Writing – original draft. LW: Conceptualization, Data curation, Formal analysis, Supervision, Writing – original draft. XL: Conceptualization, Formal analysis, Supervision, Visualization, Writing – review & editing. MS: Conceptualization, Data curation, Methodology, Software, Supervision, Writing – review & editing. LY: Conceptualization, Investigation, Project administration, Resources, Supervision, Validation, Writing – review & editing.

## Supplementary material

Supplementary Table 1 Results of the pleiotropy test.

Supplementary Table 2 Results of the heterogeneity test.

Supplementary Table 3 Results of the MRPRESSO test.
